# Distinguishing recurrence from radiation-induced lung injury at the time of RECIST progressive disease on post-SABR CT scans using radiomics

**DOI:** 10.1038/s41598-024-52828-4

**Published:** 2024-02-14

**Authors:** Salma Dammak, Stephanie Gulstene, David A. Palma, Sarah A. Mattonen, Suresh Senan, Aaron D. Ward

**Affiliations:** 1 Baines Imaging Research Laboratory, London Regional Cancer Program, London Health Sciences Centre, Victoria Hospital (A3-123A), 800 Commissioners Rd E, London, ON N6A 5W9 Canada; 2 School of Biomedical Engineering, Western University, London, ON Canada; 3 Department of Radiation Oncology, Schulich School of Medicine and Dentistry, Western University, London, ON Canada; 4 Department of Medical Biophysics, Schulich School of Medicine and Dentistry, Western University, London, ON Canada; 5 Department of Radiation Oncology, VU Amsterdam University Medical Centers, Amsterdam, The Netherlands

**Keywords:** Biomedical engineering, Computational biophysics, Cancer imaging, Radiotherapy, Machine learning, Software

## Abstract

Stereotactic ablative radiotherapy (SABR) is a highly effective treatment for patients with early-stage lung cancer who are inoperable. However, SABR causes benign radiation-induced lung injury (RILI) which appears as lesion growth on follow-up CT scans. This triggers the standard definition of progressive disease, yet cancer recurrence is not usually present, and distinguishing RILI from recurrence when a lesion appears to grow in size is critical but challenging. In this study, we developed a tool to do this using scans with apparent lesion growth after SABR from 68 patients. We performed bootstrapped experiments using radiomics and explored the use of multiple regions of interest (ROIs). The best model had an area under the receiver operating characteristic curve of 0.66 and used a sphere with a diameter equal to the lesion’s longest axial measurement as the ROI. We also investigated the effect of using inter-feature and volume correlation filters and found that the former was detrimental to performance and that the latter had no effect. We also found that the radiomics features ranked as highly important by the model were significantly correlated with outcomes. These findings represent a key step in developing a tool that can help determine who would benefit from follow-up invasive interventions when a SABR-treated lesion increases in size, which could help provide better treatment for patients.

## Introduction

Stereotactic ablative radiotherapy (SABR) is the standard treatment for early-stage non-small cell lung (NSCLC) cancer patients who are inoperable or who refuse surgery^1^. SABR is highly effective, with a five-year local control rate approximating 90%. While it is successful most of the time, for those for whom it fails, early detection of cancer recurrence is crucial. NSCLC is an aggressive disease, with a substantial drop in survival between its stages: five-year survival for stages I–II is 53–92%, while that for stages III–IV it is 0–36%, and is typically only curable in the early stages^2^. Therefore, early detection of recurrence in early-stage NSCLC cancer would allow for salvage treatment before the tumor further progresses and becomes incurable. Conversely, it is equally important to not intervene unnecessarily. Salvage therapy, such as repeat radiation or resection of a previously irradiated area, is typically more aggressive and riskier than the primary therapy; therefore, administering it to those without recurrence exposes them to the unnecessary risks of treatment-related mortality and toxicity.

However, early detection of recurrence is difficult after SABR. Like all radiation, SABR causes inflammation and fibrosis in the lung known as radiation-induced lung injury (RILI). RILI appears as an increase in density in the lungs on follow-up computed tomography (CT) scans, is largely benign and asymptomatic, and can occur in 54–100% of patients^3^. In SABR specifically, the high conformity of the radiation plan to the tumor shape results in RILI patterns that are mass-like, appearing similar to lesion growth^4^. This makes it especially difficult to distinguish RILI from cancer recurrence.

Currently, lesions with apparent growth after SABR are generally first considered by radiologists using the response evaluation criteria in solid tumors (RECIST) guidelines, where an increase in lesion size within specific criteria is considered to correspond to progressive disease. We will refer to this as “RECIST progressive disease”. On post-SABR follow-up CTs, this has low specificity, as it does not account for the apparent increase in lesion size due to RILI. In one study, 64% of SABR-treated lesions had RECIST progressive disease, when in fact only 10% of those truly recurred^5^. Nevertheless, despite its low specificity, when a lesion has RECIST progressive disease, the treating physician has to make the critical clinical decision of whether to intervene, including requesting more tests, or continue with observation alone. This is a difficult task: the average time for a physician to detect a true recurrence is 15.5 months and agreement between physicians is only moderate (k = 0.54)^6^.

Furthermore, tools that are typically available in the clinic for recurrence assessment are unable to adequately detect it in this setting. Biopsy is an invasive procedure, with 20–25% risk of pneumothorax and 41% risk of hemorrhage, both of which can lead to further complications^7^. Additionally, 48% of lung cancer biopsies are inconclusive, and conclusive negative biopsies have a false negative rate of 15%^8,9^. Using fluorodeoxyglucose (FDG) positron emission tomography (PET) is also limited. The acute inflammatory reaction in the lungs characteristic of the response to SABR increases the metabolic activity in and around the irradiated lesions, resulting in a 64% false positive rate for FDG-PET after treatment, with higher false positive rates during earlier follow-up^10^. There is therefore still a need for a method of distinguishing recurrence from RILI after SABR.

On follow-up CT, physician-scored high risk features (HRFs), such as an enlarging lesion at or after 12 months, bulging margin, and cranio-caudal growth, have been shown to correlate with recurrence^11^. However, these features may not always develop with recurrence or may develop after RECIST has been triggered, limiting the ability to use them at this critical decision point. Radiomics features have been shown to predict recurrence, making them an attractive approach for this problem^6,12^. Mattonen et al. established that radiomics can predict recurrence from the three-month follow up CT scan^6^. However, their results are likely to be optimistically biased as the entire dataset was used when selecting features. Furthermore, the dataset was relatively small and did not include an independent test set. Kunkyab et al*.* proposed a model trained on later scans to predict early recurrence in a test set, but their model still needs to be validated on patients whose scans were not used for developing the model^12^. Consequently, there is still a need for a model that is built and validated for the clinical decision point, which is when the lesion has RECIST progressive disease.

Our overarching goal therefore is to use radiomics to build a model that can distinguish RILI and recurrence at the time of lesion growth after SABR (i.e., RECIST progressive disease) and to try to uncover features that are important for this distinction. To this end, the key questions we address in this paper are as follows:

1. What is the performance of a radiomics-based model for distinguishing recurrence from RILI at the time of RECIST increase on a CT scan after SABR when considering
  - *Different ROIs*, when different ROIs are used for radiomics calculation;
  - *Inter-feature correlation*, when highly correlated features are removed compared to when they are included; and
  - *Volume correlation*, when volume correlated features are removed compared to when they are included?
2. *Feature analysis:* what radiomics features are most important for such a model?

## Methods

### Study Sample

We obtained retrospective pre-treatment and follow-up CT scans from 68 patients with a single lung tumor and early-stage NSCLC who were treated with SABR at the VU Amsterdam University Medical Centers (VUMC) in the Netherlands and had RECIST progressive disease on follow-up CT scans. A radiation oncologist determined the lesion’s longest axial diameter by RECIST 1.1 criteria on all CT scans, as would be done during the typical clinical workflow^13^. We then used these RECIST lines to determine which follow up CT scan corresponds to RECIST progressive disease for each patient. RECIST progressive disease is an increase of ≥ 20% in the length of a lesion’s longest axial diameter from its smallest size, including its size prior to treatment, that is also at least 5 mm in absolute length. For each patient, we only used this scan for subsequent analysis, as this corresponds to the critical clinical decision time point.

The follow-up CT scans were taken at inspiratory breath hold, with 70 cc of intravenous contrast (Ultravist-300; Bayer Pharma AG, Berlin, Germany) administered with a 25 s delay. This was done on either a Siemens Volume Zoom 4-slice, Siemens Sensations 64-slice (Siemens Nederland N.V., Den Haag, Netherlands) or a Philips Brilliance iCT 256-slice (Royal Philips Electronics, Inc., Amsterdam, Netherlands).

The outcomes of recurrence or RILI were determined based on one or a combination of: biopsy (10%), FDG-PET (12%), later CT scans (29%), and subsequent growth on CT imaging (68%).

The University of Western Ontario Health Sciences Research Board is the ethics board of record for this project and provided approval and ongoing annual monitoring (project number #105444). The collection and analysis of this data were approved by this board and completed as per its guidelines. Patient informed consent was waived by the ethics board of The University of Western Ontario Health Sciences Research, as per its guidelines, patient consent is not required for this retrospective study.

### Regions of interest (ROIs)

**Figure 1:**
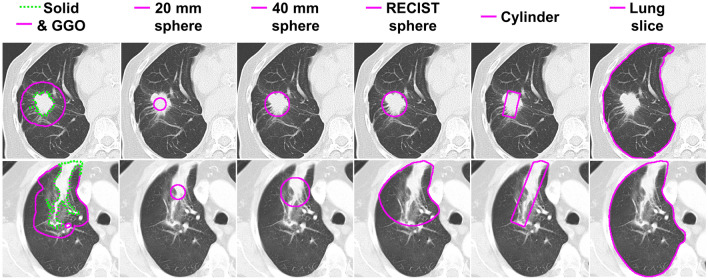
An axial slice through the six regions of interest we tested in our study for two tumors, one on each row, shown on the CT scan [window: 1500 HU, level: − 600 HU] with RECIST recurrence.

The semi-automatic delineation of lung lesions post-SABR has been shown to perform similarly to manual delineation for recurrence prediction^12,14,15^. In this study, we aim to investigate this further through studying various ROI designs to determine which is best for this problem. To do this, we conducted our analysis within six ROIs, shown in Fig. 1, all of which were restricted to the lung volume. The volumes of both lungs were delineated semi-automatically in ITK-SNAP 3.8.0 using region competition snakes following the protocol described in a previous publication by Mattonen et al*.*^15,16^. Each region of interest was initialized using a RECIST line drawn by the radiation oncologist. These were delineated by a radiation oncologist using the ruler tool in ClearCanvas Workstation 2.0 (Synaptive Medical, Toronto, Canada) and exported for subsequent use in MATLAB 2019b (The MathWorks Inc., Natick, MA, USA) using the RulerPlus module we developed in-house.

Most of the ROIs we analyzed were three-dimensional (3D). The solid and ground glass opacity (GGO) ROI consists of two sub-ROIs, where the solid sub-ROI was designed to approximate the solid component of the lesion, and the GGO sub-ROI was designed to capture tissue surrounding the solid including any ground-glass-like opacification in the surroundings of the solid. This ROI was originally proposed by Mattonen et al*.* and shown to represent manual contours of these components well^15^. We obtained it as previously described by them^15^. First, we obtained the solid sub-ROI using the OneCut algorithm with the RECIST line acting as foreground and a ring circumscribing the RECIST line acting as background^17,18^. We then obtained the GGO sub-ROI by using a 16 mm concentric expansion of the solid volume.

As a way to attempt simplifying the geometrical representation of the solid and GGO ROI, we studied the use of a sphere centered on the RECIST line midpoint. Here we studied various sphere diameters: 20 mm, 40 mm, and the RECIST line length for the lesion. These ROIs can include appearance features from both the lesion and its surrounding lung parenchyma, with varying amounts of each based on the sphere size and the size and shape of the lesion.

Additionally, we used a cylinder-shaped ROI with the RECIST line as its rotation axis, with a 10 mm radius. This was designed to allow us to sample the lesion appearance immediately around the RECIST line, which would exclude most surrounding lung parenchyma. This would allow us to understand how well the lesion tissue alone and not the tissue surrounding it, can predict recurrence.

One of the ROIs we studied was a two-dimensional (2D) lung slice and consisted of the segmented lung visible in the axial slice where the RECIST line was drawn, which corresponds to where the lesion was largest axially. We chose to investigate this ROI because it would be the easiest to translate clinically, as it only requires the lung contour, which is relatively easy to obtain, and that the physician selects the slice where the lesion has the longest axial diameter.

### Machine learning

We performed image pre-processing and radiomics feature extraction using an in-house extraction library built in MATLAB 2019b (The MathWorks Inc., Natick, MA, USA). The CT scan voxel dimensions ranged as follows: 0.57–0.84 × 0.57–0.84 × 1.50–5.00 mm^3^. To ensure that subsequent radiomics feature calculations were meaningful spatially across scans, we resampled all images to the most common (54% of scans) voxel dimensions of 0.74 × 0.74 × 5.00 mm^3^.

For all ROIs, we calculated 20 first order texture features, 21 gray level co-occurrence matrix (GLCM) features, and 11 gray level run length matrix (GLRLM) features using the feature extraction parameters in Table S.1 of the supplemental materials. We calculated the GLCM and GLRLM in the four unique neighboring directions in the axial plane, then averaged over all directions, resulting in five direction options per feature, and a total of 180 features. We excluded out-of-plane neighbors due to the high voxel anisotropy in the CT scans in that direction. For the solid and GGO ROI, we calculated the 180-set of features separately in each sub-ROI, then combined the two sets of features such that the model would be free to choose features from both sub-ROIs. For the solid sub-ROI, we also calculated 24 shape features, as this has been shown to be important for recurrence prediction in previous work^19^. This resulted in a total of 384 features for the solid and GGO ROI.

We used each ROI’s set of features as input to an identical experiment algorithm also built in-house in MATLAB 2019b (The MathWorks Inc., Natick, MA, USA), based on the implementation by Devries et al*.*^20^. In this algorithm, we used 500 iterations of bootstrapped resampling with replacement to split the dataset randomly into the training and testing datasets. The training set size was set to the full dataset size (n = 68), as is typical in bootstrapping experiments, and the testing set size was of variable size as it contained the unique samples not used in training, which varied in number across iterations. On average, the training set had 43 unique samples (63.2%) and the testing set had 25 unique samples (36.8%), as is expected for sampling with replacement. We used the training dataset first to build an inter-correlation filter, where we calculated the Pearson correlation between all possible features pairs. When the correlation for feature pair was > 0.80, we eliminated the feature that is less correlated with the outcome labels in the training set. We also used the training set for hyper-parameter optimization and training of a random forest model. The model hyperparameters are in Table S.2 in the supplementary materials.

On the test set, we obtained the predicted probabilities from all bootstrap iterations and aggregated them to calculate an average receiver operating characteristic curve (ROC), its area under the curve (AUC), and their associated 95% confidence intervals (CIs). We also calculated sensitivity and specificity and their 95% CIs. To determine the ROC operating point at which to calculate them for testing, we aggregated the predicted probabilities on the training set out-of-bag samples for each tree in the random forest, built an ROC curve from them, and used the operating point corresponding to the upper left corner of this out-of-bag training set curve for the testing set.

### Experiments addressing each research question

To answer Q1a, where we aimed to determine the effect of ROI on model performance, we compared the test AUCs across all ROIs. To do this, we first calculated the AUC for each bootstrap iteration, obtaining a set of 500 AUCs per ROI. We then performed a one-sample Kolmogorov–Smirnov (KS) test to test for normality. If all sets of AUCs were normal, we performed a one-way analysis of variance (ANOVA), otherwise we performed a Kruskal–Wallis test. We then performed post-hoc tests with the Bonferroni correction, using average ranks if not normal, to determine which ROIs were significantly different from each other. We used an α = 0.05 for all statistical tests in this paper.

In Q1b, our aim was to determine the effect of removing inter-correlated features on model performance. To test this, we selected the ROI with the highest average AUC from the previous question and repeated the experiment for it without the inter-correlation filter. We then calculated the AUC for each iteration of the experiment, with and without the inter-correlation filter, obtaining a set of 500 AUCs for each. If both sets were normal, as determined through the KS test, we used a t-test to test if they were significantly different, otherwise, we used a Wilcoxon Rank-Sum test.

For Q1c, our aim was to determine if tumor size had an effect on performance. To test this, we first selected the model with the highest AUC up to this point. We then calculated the volume of the ROI as an approximation of tumor size. While ROI volume does not directly measure the effect of tumor volume, it does so indirectly as all ROIs vary in size in correlation with the RECIST line length, except for the 20 mm and 40 mm spheres, and if the latter two were chosen before this point it would immediately indicate that volume is not correlated with performance. From there, we determined which features were correlated with volume in the training set, and removed any that had a significant Pearson correlation coefficient that was > 0.5. Then, as we have done previously, we calculated the AUCs for every iteration with and without the volume correlation filter, and used a t-test to compare the two options if the distributions were both normal, and the Wilcoxon Rank-Sum test otherwise.

To understand if tumor size had an inherent correlation to the outcomes for our whole dataset, we also calculated the correlation coefficient between the volume and the outcomes, and the RECIST line size and the outcomes. For both, we tested for normality using the KS test, and used the Point-Biserial correlation coefficient when normal, and Rank-Biserial correlation when not.

To answer Q2, where we aimed to investigate what features are most important for this problem, we selected the model with the highest AUC up to this point. From there, we calculated feature importance rankings, inherent to how random forest models are built. To do this for all bootstrap iterations, we first normalized the feature importance scores between 0 and 1 for every bootstrap iteration and set any features that were removed by a correlation filter, if any, to 0. We then averaged these scores across iterations and normalized the averaged scores between 0 and 1 again. We then used a threshold of > 0.80 to find which features were most important on average across bootstrap iterations.

As a way of estimating the importance of each of these individual features for this problem, we measured their correlation with the outcome for the whole dataset. For normally distributed features, we used a Point-Biserial correlation, otherwise we used a Rank-Biserial correlation. Lastly, as a way of comparing the features in terms of their likelihood to perform well, we built an ROC based on each feature and calculated its AUC. Here, we normalized the feature values between 0 and 1 and used them as the confidences for the ROC. Also, if any feature was anti-correlated with outcomes, we flipped the outcomes to accommodate this. Lastly, we calculated sensitivity and specificity at the operating point in the upper left corner to gain a better understanding of the feature’s performance.

## Results

The median patient age in this study was 72 years (range of 57–88 years), 33% of patients were female, and SABR treatments consisted of 54–60 Gy in 3–8 fractions. The overwhelming majority of patients in our study (98%) triggered RECIST progressive disease in the first year after treatment, with 25% triggering RECIST progressive disease at first follow-up scan, 38% at the second, 25% at the third, and 12% at the fourth scan. These correspond approximately to three, six, nine, and twelve months post-treatment, respectively, although the actual timing of the scans was subject to normal clinical variability. Of all these patients, only 41% were later determined to have a true recurrence. Note that the percentage of recurrences were higher in our dataset than the expected approximate 10% because our sample was originally enriched for patients with recurrence.

### Effect of ROI on performance

As shown in Table 1, all ROIs resulted in models with average AUCs, including their 95% CIs, above 0.50. This shows that given this model design, any of the ROIs can be used to build a model that can distinguish recurrence and RILI at the time of RECIST progressive disease with a performance that is better than chance. Furthermore, most ROI pairs did not have significantly different AUCs, indicating that they may be interchangeable from a performance perspective. However, an important exception to this was the RECIST sphere which had an AUC higher than and significantly different from all other ROIs, with the biggest difference in AUC being 0.06. The RECIST sphere also had a higher specificity than all other ROIs, with no overlap in specificity 95% CIs between it and the other ROI. This was not the case for sensitivity, which signals that this ROI’s higher AUC is driven by improved specificity. The other ROI that was significantly different from other ROIs was the lung slice ROI, which had a lower than and significantly different AUC from the sphere-based ROIs. This means that, given the choice between the lung slice ROI or any of the sphere ROIs, the sphere ROIs would be superior in terms of performance.

**Table 1:** Test set performance (95% CIs in brackets) based on the average ROC across 500 bootstrap iterations given various regions of interest as input.

ROI	AUC [95% CIs]	Sensitivity [95% CIs]	Specificity [95% CIs]
Lung slice	0.58 [0.57, 0.58]^¥^	57% [56%, 59%]	55% [53%, 56%]
Solid and GGO approximation	0.58 [0.57, 0.59]	56% [55%, 58%]	53% [52%, 55%]
Cylinder	0.59 [0.58, 0.59]	55% [53%, 56%]	59% [57%, 60%]
Sphere (d = 40 mm)	0.59 [0.58, 0.60]	52% [50%, 53%]	60% [59%, 62%]
Sphere (d = 20 mm)	0.60 [0.59, 0.61]	57% [56%, 59%]	57% [55%, 58%]
Sphere (d = RECIST length)	0.64 [0.63, 0.65]*	57% [56%, 59%]	65% [64%, 67%]

*The RECIST sphere ROI AUC is significantly different than all other ROIs (*p* ≤ 2.1 × 10^–8^).

^¥^The lung slice ROI is also significantly different from the 40 mm (*p* = 0.012) and 20 mm (*p* = 0.0024) sphere ROIs.

### Inter-feature correlation

To test the model performance for the inter-feature correlation filter, we used the RECIST sphere ROI as that had the highest AUC up to this point. With the filter, the model’s AUC was 0.01 lower than without it, and this difference was statistically significant, as shown in Table 2. This indicates that using the filter is detrimental to model performance.

**Table 2:** Test set performance (95% CIs given in brackets) based on the average ROC across 500 bootstrap iterations with and without the use of correlation filters. This was using the RECIST sphere. Note that the first row was already shown in Table 1 (last row) and copied here for ease of comparison.

Inter-feature correlation filter	Volume correlation filter	AUC [95% CIs]	Sensitivity [95% CIs]	Specificity [95% CIs]
yes	No	0.64 [0.63, 0.65]*	57% [56%, 59%]	65% [64%, 67%]
No	No	0.65 [0.65, 0.66]	59% [57%, 60%]	65% [64%, 67%]
No	Yes	0.66 [0.65, 0.67]	59% [57%, 60%]	66% [65%, 68%]

*The first row is significantly different from both the second (*p* = 0.013) and third (*p* = 0.0010) rows.

### Volume correlation

To test the effect of removing volume-correlated features prior to training the model, we used the RECIST sphere ROI without the inter-feature correlation filter, as this combination had the highest performance up to this point. Using the volume correlation filter led to an AUC that was 0.01 higher on average than not using it, as shown in Table 2, but this difference was not significant (*p* = 1.00). This indicates that removing volume-correlated features has no significant impact on model performance. Furthermore, neither volume nor RECIST line length were significantly correlated with the outcome for the whole dataset. Volume had a correlation of 0.021 (*p* = 0.87) while RECIST line length had a correlation of 0.078 (*p* = 0.53). This indicates that that a relationship does not exist between RECIST sphere volume nor RECIST line length with outcomes in our dataset.

Note that as our dataset was enriched for patients with recurrence, the label distribution within it did not match the population level of recurrence. To evaluate whether this affected performance, using the best model (i.e., RECIST sphere ROI, no inter-feature correlation filter, with volume correlation filter), we decimated the test set in every bootstrap iteration to make the percentage of positives as close as possible to 10%, which is the population percentage. The median AUC across these modified test sets was 0.66 with [0.64, 0.68] 95% CI. This was not significantly different from the AUC obtained on the original test sets, but the 95% CI was slightly wider, which is to be expected with the reduction in overall dataset size driven by the decimation.

### Feature analysis

To perform feature analysis, we analyzed the results of model with highest performance up to this point. This used the RECIST sphere ROI, did not use the inter-feature correlation filter, and used the volume correlation filter. Features that were highly important (i.e., average feature importance score > 0.80) as ranked by this model are listed in Table 3 in order of importance. As shown in this table, all features selected by this model had a significant correlation with outcomes, with absolute correlation coefficient values ranging from 0.26 to 0.31. Furthermore, all features had an AUC better than chance for distinguishing the recurrence from RILI for the whole dataset when the normalized feature values were used as classification confidences. This shows that these features were indeed important for distinguishing the two outcomes in our dataset.

**Table 3:** The top features across 500 bootstrap iterations for the model with the highest AUC. The correlation coefficient is the Rank-Biserial correlation coefficient. The AUC was obtained by normalizing the feature values between 0 and 1 and using them as the confidences, and the sensitivity and specificity were recorded at the operating point corresponding to the upper left corner of the curve.

Name	Equation	Offset^†^	Correlation coefficient	*p*-value	AUC	Sensitivity (%)	Specificity (%)
Skewness	$\frac{{mean\left(I\right)}^{3}}{{stdev\left(I\right)}^{3}}$	–	− 0.26	*0.032*	0.70*	65	82
GLCM max	${\text{max}}\left(GLCM\right)$		0.38	*0.0015*	0.56	36	98
GLCM joint energy	$\sum {GLCM}^{2}$		0.30	*0.013*	0.53	32	93
Mean	$mean\left(I\right)$	–	0.31	*0.011*	0.68	68	80

*As skewness is anti-correlated with outcome, the outcomes were reversed for the AUC calculation.

$I$: all voxel intensity values within the ROI. GLCM: Grey Level Co-occurrence Matrix.

^†^The offset refers to the direction of the GLCM calculation. In the diagrams provided in this column, the center grey squares denote the current pixel of interest while the black squares denote the neighbors of that pixel that are being considered.

Significant values are in italic.

## Discussion

In this paper, we have shown that radiomics can distinguish recurrence and RILI better than chance on post-SABR follow-up CT scans where the lesion had clinically meaningful growth, i.e., had RECIST progressive disease. To the best of our knowledge this is the first paper to present a model for distinguishing the two outcomes on standard-of-care CT scans at this critical clinical decision time point, when standard metrics (i.e., RECIST) indicate possible progressive disease. We have shown that this is possible for several ROIs that are obtained semi-automatically, but that the choice of ROI significantly affects model performance, with a sphere that uses the RECIST line as its diameter to be the best-performing ROI option. We have also shown that removing features that are correlated with each other affects performance, but to a smaller extent than the choice of ROI.

Additionally, we have found that removing features that are correlated with ROI volume does not affect performance significantly, and that the outcomes in our dataset are neither correlated with ROI volume nor with RECIST line length. This is an important finding, as the increase in RECIST line length is currently the most commonly used tool in the clinic to detect recurrence, and its lack of correlation with outcome for patients with apparent lesion growth shows how limited it is for predicting cancer recurrence. This highlights the need for a tool that can aid physicians at this decision point.

Lastly, for the best-performing model in this paper, we found that four features were ranked as highly important by the model: skewness and mean, and GLCM maximum and joint energy. In subsequent analysis, we found that these four features had a significant correlation with the outcomes in our dataset, and an AUC above chance when their normalized feature values were used to predict outcomes for the whole dataset. This confirms that these features are indeed important for distinguishing RILI and recurrence in this dataset.

### Effect of ROI on performance

While all ROIs led to a model that had an AUC above chance for distinguishing recurrence from RILI, the RECIST sphere ROI performed best. The only difference between this ROI and the other sphere ROIs is that the RECIST sphere varied in size with the axial size of the lesion. This meant that for every lesion, this ROI fully encompassed the parts of the lesion on the RECIST line plus additional tissue that was a mix of lesion and its surrounding tissues, maximizing lesion coverage and capturing both surrounding GGO and normal lung parenchyma. In comparison, the design of the other two spheres did not guarantee that the RECIST line was fully encompassed. In fact, the average RECIST line length in our dataset was 55 mm, which meant that the 40 mm and 20 mm spheres would have frequently missed parts of the lesion. This is also likely the case for the cylinder, which would have missed large parts of the lesion if the lesion was not primarily oblong, and the lung slice, which could not encompass a 3D lesion as it was 2D. It is possible therefore that the RECIST-shaped sphere performed better than these ROIs because it had more lesion tissue, and therefore more information.

However, if full lesion coverage was the most important aspect, the solid and GGO ROI would be expected to perform best, yet this was not the case. The biggest difference between this ROI and the RECIST sphere is that the margins of the lesion could be analyzed in the latter but not the former, as the solid and GGO ROI split the lesion at a point along the margin to obtain its sub-ROIs. Several HRFs are associated with the appearance of lesion margins, such as bulging and linear margin, and it is therefore plausible that allowing the model to analyze and use margin appearance led to this observed superior performance.

### Inter-feature correlation

Removing inter-correlated features led to worse performance than when the features were included, which was unexpected. However, there are two potential explanations for this. A decision tree uses features in a non-linear way, and it is possible that the feature that was removed had a better correlation with outcomes, but that that correlation was not linear, which may be discovered by a decision tree. Furthermore, decision trees combine features instead of using them individually, and while one feature of the pair had a worse correlation with outcomes individually, and was therefore eliminated, it may have resulted in a highly separable feature space when combined with other features. For example, one could conceive of a theoretically possible instance where two features build a feature space where one class is distributed in a circle and the other class is distributed in a ring around that circle. Each of these features individually would not have good correlation with outcomes, but when used together a decision tree can separate the classes perfectly as shown in Fig. S.1. in the Supplementary Materials. It is possible therefore that using a feature correlation filter inadvertently eliminated effective combinations of features such as this one.

### Volume correlation

Previous work in the literature has shown that radiomics features can be dependent on lesion volume^21^. However, in this paper we have found that the model is not directly dependent on volume: we found that removing volume correlated features did not change performance significantly and that neither ROI volume nor RECIST line length had a significant correlation with outcomes. This highlights the inadequacy of size-based measured for outcome prediction when patients have RECIST progressive disease, and further reinforces that the use of RECIST lines as a primary tool for predicting recurrence is not sufficient in the clinic. This is in line with previous findings in the literature^22^.

### Feature analysis

**Figure 2:**
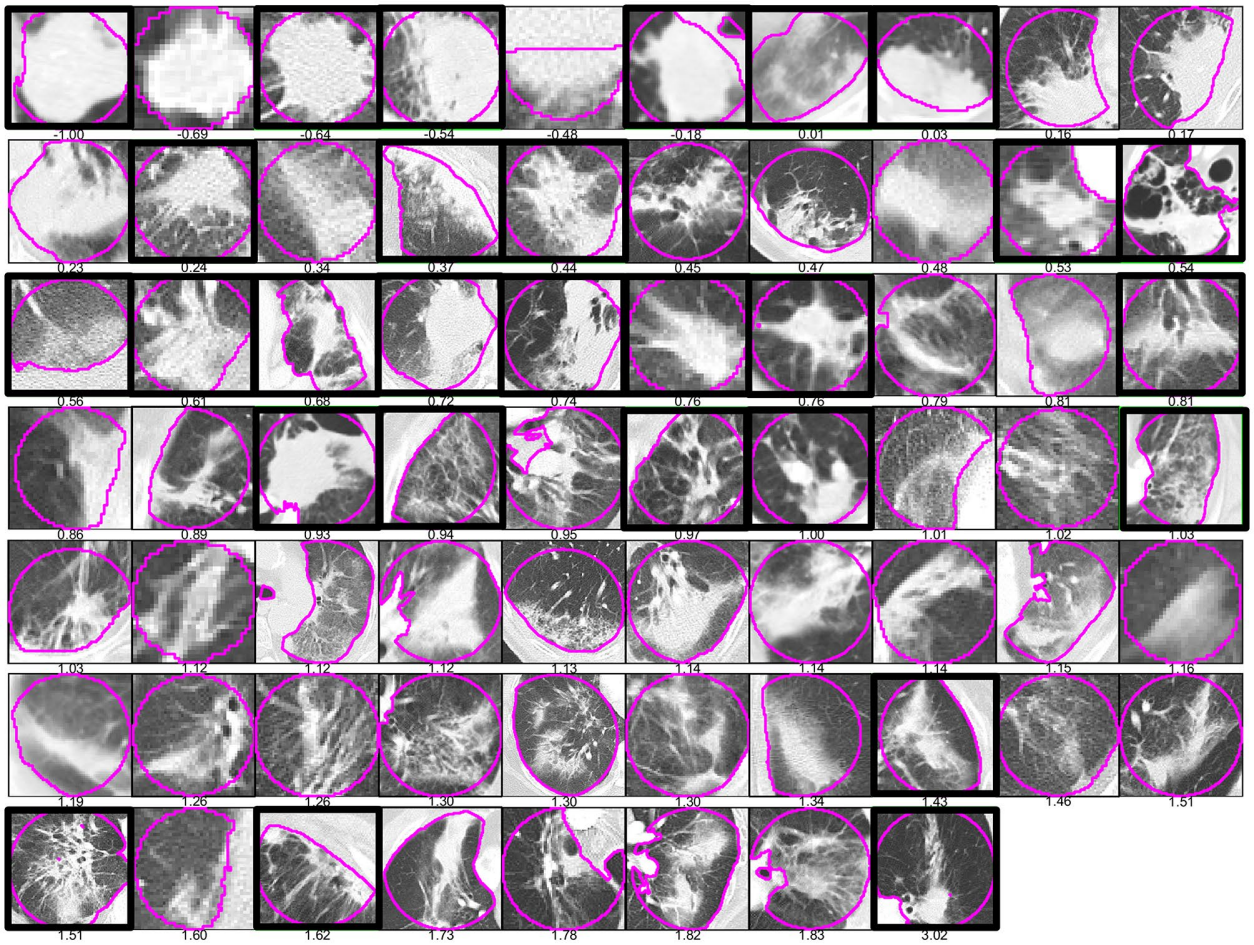
A slice through the RECIST sphere ROI of each patient in this dataset ordered by skewness values, with recurrences indicated with a thick black border. The images were scaled to allow the ROI to be fully visible in that slice (i.e., images can be of different sizes), and a window of 1500 HU was used with a level of -600 HU.

On qualitive analysis of the ROIs, ordered by feature value, an example of which is shown in Fig. 2, we observed that lower skewness values appeared to be more likely to correspond to lesions with bulging margin. Low skewness is correlated with recurrence as shown by its negative correlation coefficient value, as is bulging margin^11^. By comparison, higher values of this feature appeared correlated with the linear margin HRF or linear growth in general, which is associated with non-recurrence^11^. This shows that skewness may be capturing HRFs at a point where they are too subtle for detection with the human eye.

High values of both GLCM Max and GLCM Joint Energy, which correspond to a prediction of recurrence, appeared to correlate with mass solidity and homogeneity. This is shown in Figs. S.2 and S.3 in the Supplementary Materials. Likewise, the mean appeared to capture lesion density, with denser lesions having high mean values, shown in Fig S.4 in the Supplementary Materials. This is consistent with HRFs, where recurrent lesions are characterized by an increase in density and loss of air bronchogram, both of which would result in the appearance of a more homogenous lesion as captured by the GLCMs and more dense lesion as captured by the mean^11^. Interestingly, this corresponds to different HRFs than what skewness may be capturing, which suggests that these features could work synergistically to result in a stronger overall signature than any of the features alone.

### Limitations

An important limitation of this work is that the best test model performance is modest, which limits its clinical useability as a stand-alone tool to determine local recurrence. However, current clinical tools tend to have high sensitivities but low specificities (e.g., specificities of 35% for RECIST progressive disease and 36% for FDG-PET) while the tool we propose here has relatively high specificity (i.e., 66%)^5,10^. This means that it can work synergistically with pre-existing clinical tools to enhance the overall prediction of local recurrence. We envision this aiding in first determining whether biopsy is worth the risk, and then using the biopsy results to determine if salvage treatment is necessary.

Another limitation of this study is that it is a single-center study, thus, we cannot reliably estimate how our findings would translate on data from another center. However, this is the first study that presented a model for predicting lung cancer recurrence post-SABR on scans where lesions had apparent growth and acts as a pilot study for this problem. In future work, we aim to address this by testing this model and this approach on data from another center.

This would also address a different limitation. By finding a feature signature across bootstrap iterations, using those that were ranked as highly important by the model across iterations, we effectively used the whole dataset to determine the feature signature. This made it such that the signature was not tested on unseen data. This limits our ability to predict how well it is likely to perform on new data, and by testing it on an additional dataset, we can verify this feature signature’s ability to generalize.

Another limitation is that we used a random forest model. These models are very powerful, but they are hard to interpret and to make generalizable. This complexity is further amplified by the use of bootstrapping, as this does not allow us to obtain a single model at the end of the experiment. However, this methodology allowed us to robustly identify a much smaller feature set that is important for this problem, which would allow us to use a less complex model (e.g., a single decision tree) in future work. This pilot study therefore paves the way for building a more robust and generalizable model in the future.

Lastly, in this study, we used biopsy, FDG-PET, and subsequent CT follow-up to determine the ground truth labels, all of which carry a degree of inaccuracy. Biopsy is the most accurate, with perfect precision and 15% miss rate^9^. However, it is not always feasible due to tumor inaccessibility or increased biopsy risk, both of which typically motivate the use of SABR instead of surgery, and were therefore often encountered for our study population. The next best option is FDG-PET, as when it is obtained at least one year after SABR, it can discriminate between RILI and recurrence with a sensitivity and a specificity of 91% or higher^11^. Serial CT examination is the next best option. On CT scans taken at least one year after treatment, specialist radiologists and radiation oncologists have a median sensitivity of 83% and median specificity of 75% for distinguishing RILI from local recurrence^6^.

Due to the imperfect accuracy of these methods, the ground truth labels in our study likely carry some noise. This would have likely resulted in suboptimal model training, as noisy labels would have made it more difficult for the model to detect the patterns associated with each label. We would therefore expect to see improved performance if a more accurate method of obtaining ground truth labels is used. We do not, however, expect this to have much effect on the reliability of our test results, as we used bootstrap resampling. By randomly resampling the test set and using the average performance, we could ensure that test performance is minimally affected by any specific patients, including the small subset of the patients in our dataset that could hypothetically have noisy labels.

## Conclusion

In conclusion, we have shown in this study that a radiomics-based model can distinguish RILI and recurrence on an unseen test set with an average AUC of 0.66 (95% CIs [0.65, 0.67]), average sensitivity of 59% (95% CIs [57%, 60%]), and average specificity of 66% (95% CIs [65%, 68%]) across 500 bootstrap iterations. We found that the choice of ROI significantly affects performance, with a RECIST-line-sized sphere as the best-performing ROI, but that all ROIs that we tested had an AUC ≥ 0.58 (95% CIs [0.57, 0.58]). We also found that removing inter-correlated features prior to training the model negatively affects performance (*p* = 0.013) but that removing volume correlated features had no significant effect on it (*p* = 1.00). Indeed, we found that there was no correlation between volume and outcome (*r* = 0.021, *p* = 0.87), further highlighting the need for a tool to distinguish RILI from recurrence that does not rely on volume. In analyzing the features most commonly used by the best-performing model, we found that two first order features: skewness and mean, and two GLCM features: maximum and joint energy, were rated as most important. On follow-up analysis, we found that all features were significantly correlated with outcomes (*p* ≤ 0.032), and on qualitive analysis, we saw that the feature values tended to correspond to HRFs. With further development, a model using these features could act as a decision support tool that helps physicians determine whether a patient with early-stage lung cancer has RILI or recurrence when the tumor appears to recur on CT. This could help identify patients who need salvage therapy at this critical clinical decision point and could help spare those with RILI the risk of toxicity from unnecessary treatment.

### Supplementary Information

Supplementary Information.

## Data Availability

The predicted probabilities, ground truth labels, and dataset split indices for each experiment described in this study are available and can be used to replicate the study’s analyses at: https://github.com/SalmaDammak/PostSABRRecurrenceOnRECIST

## Code availability

All code used to generate the study’s results is available at: https://github.com/SalmaDammak/PostSABRRecurrenceOnRECIST
