# Supplementary material for: Distinguishing recurrence from radiation-induced lung injury at the time of RECIST progressive disease on post-SABR CT scans using radiomics
Source: Sci Rep. 2024 Feb 14;14:3758. doi: 10.1038/s41598-024-52828-4 (PMC10866960; doi:10.1038/s41598-024-52828-4)
Supplement: Supplementary file 1 — Supplementary Information. [file 41598_2024_52828_MOESM1_ESM.pdf]

Table S.1: Radiomics feature extraction parameters.

	Parameter	Value
**General**	Minimum number of voxels per ROI	4
**GLCM features**	First bin Edge	-1000
	Bin size	20
	Number of bins	110
	Look-up distance	1
**GLRLM features**	First bin edge	-1000
	Bin size	20
	Number of bins	60
	Run threshold	5
	Number of columns	Max Image Volume Dimension
**First-order features**	Number of bins for entropy/uniformity	20

Table S.2: Random forest classifier parameters. Hyperparameter optimization was done using the training set for the parameters marked as "optimized".

Parameter name	Value	Optimization domain
Number of trees	optimized	[10,1000]
In-bag fraction	100%	NA
Sample with replacement	'on'	NA
Number of predictors to sample	optimized	[1, number of features]
Minimum leaf size	optimized	[1, number of samples/2]
Maximum number of categories	10	NA
Maximum number of splits	optimized	[1, number of samples-1]
Merge leaves	off	NA
Prune	off	NA
Split criterion	optimized	[Gini's Diversity Index, Deviance]

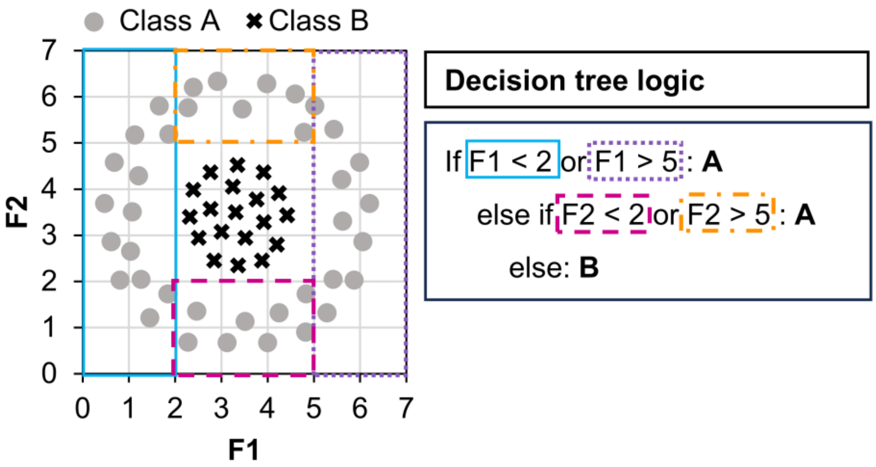

Figure S.1: Theoretically possible sample distribution where individual features would have poor performance separating classes individually using a decision tree but perfect performance separating them as a combined pair.

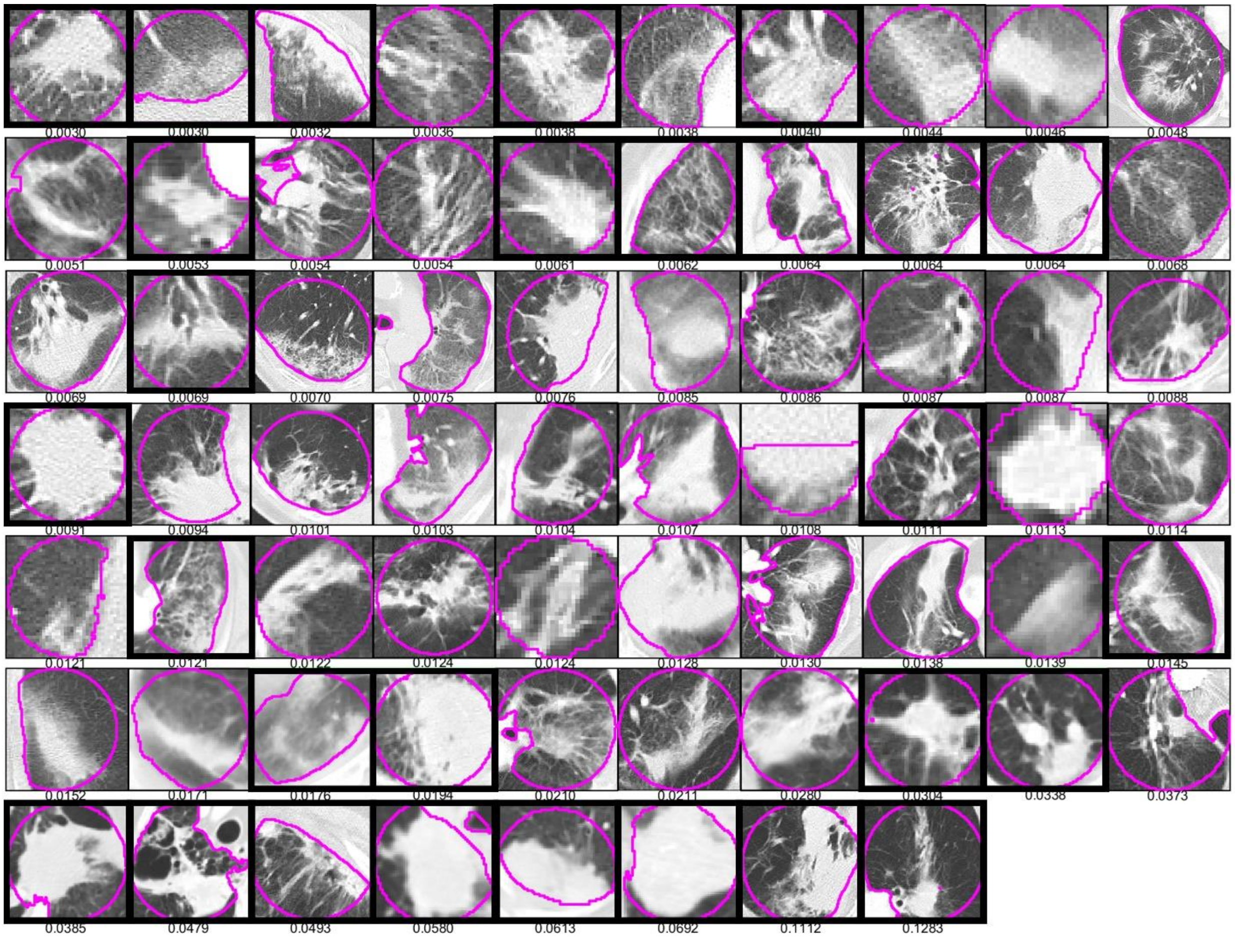

Figure S.2: A slice through the RECIST sphere ROI of each patient in this dataset ordered by GLCM Max values, with recurrences indicated with a thick black border. The images were scaled to allow the ROI to be fully visible in that slice (i.e., images can be of different sizes), and a window of 1500 HU was used with a level of -600 HU.

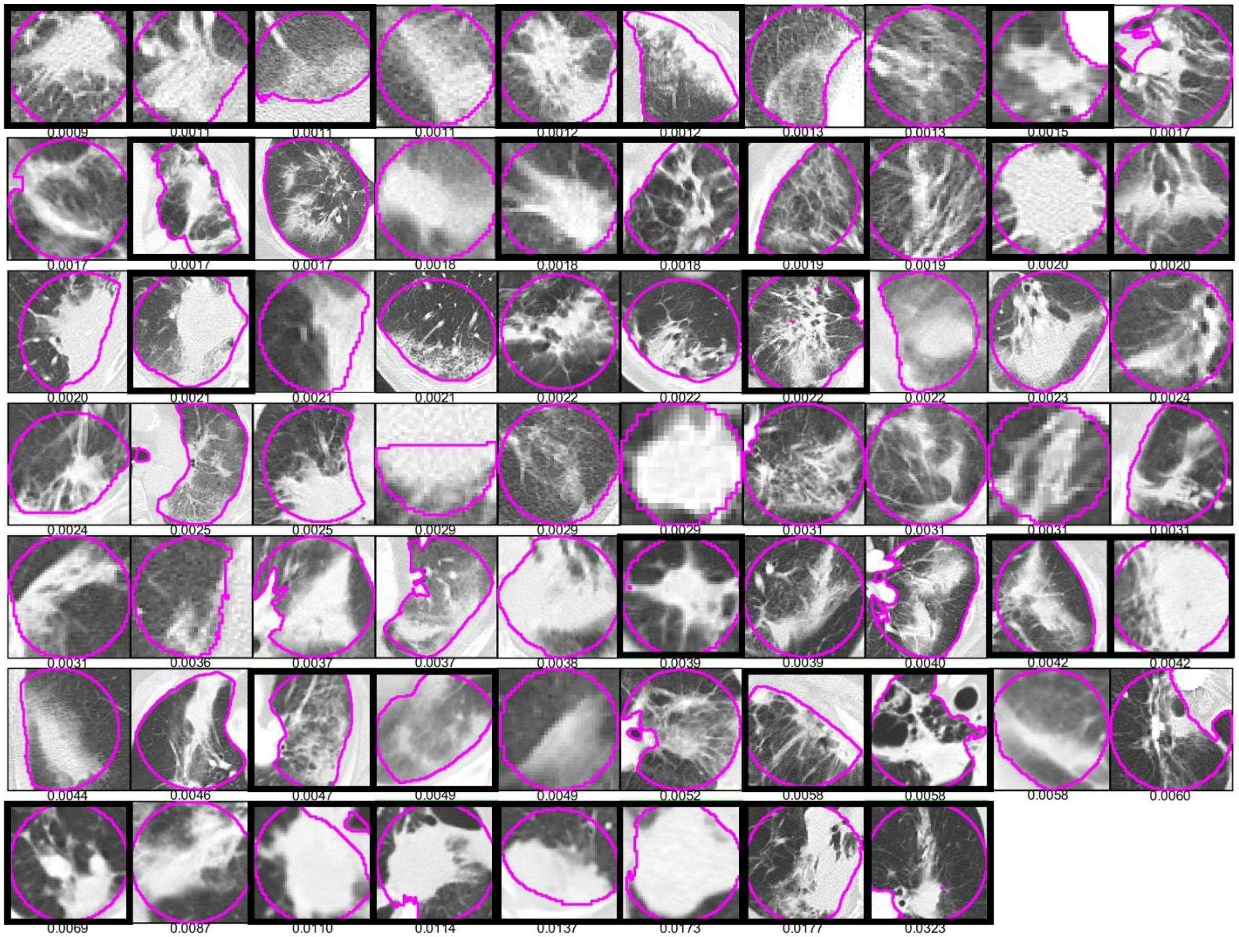

Figure S.3: A slice through the RECIST sphere ROI of each patient in this dataset ordered by GLCM Joint Energy values, with recurrences indicated with a thick black border. The images were scaled to allow the ROI to be fully visible in that slice (i.e., images can be of different sizes), and a window of 1500 HU was used with a level of -600 HU.

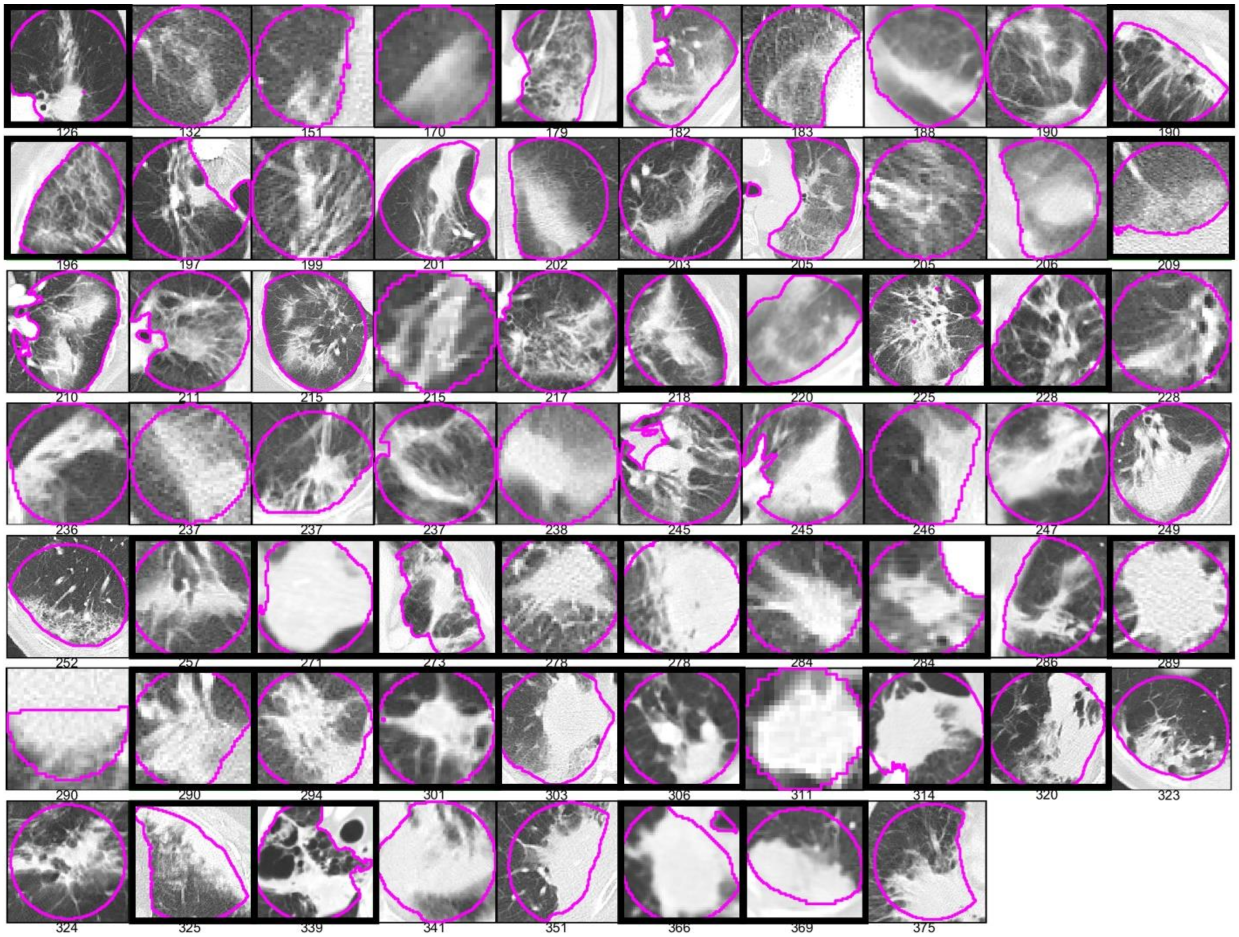

Figure S.4: A slice through the RECIST sphere ROI of each patient in this dataset ordered by mean intensity values, with recurrences indicated with a thick black border. The images were scaled to allow the ROI to be fully visible in that slice (i.e., images can be of different sizes), and a window of 1500 HU was used with a level of -600 HU.
